# Assessment of the brain impact of soccer heading using pupillary light reflex

**DOI:** 10.3389/fneur.2025.1603033

**Published:** 2025-05-27

**Authors:** Junzo Nakao, Ai Muroi, Aiki Marushima, Kuniharu Tasaki, Yoshiaki Inoue, Yuji Matsumaru, Eiichi Ishikawa

**Affiliations:** ^1^Department of Neurosurgery, Institute of Medicine, University of Tsukuba, Tsukuba, Japan; ^2^Department of Emergency and Critical Care Medicine, Institute of Medicine, University of Tsukuba, Tsukuba, Japan; ^3^Department of Ophthalmology, Institute of Medicine, University of Tsukuba, Tsukuba, Japan

**Keywords:** brain impact, heading, pupillary light reflex, soccer, traumatic brain injury

## Abstract

**Background:**

Soccer heading is linked to adverse cognitive effects and changes similar to traumatic brain injury (TBI). In recent years, pupil light reflex (PLR) analysis via pupillometry offers a practical, reliable and objective neurological assessment for TBI. This is the first study to evaluate brain impact from soccer heading by evaluating PLR. We aimed to evaluate changes in PLR from heading and investigate if rubber balls reduce brain impacts compared with regular soccer balls.

**Methods:**

Our study involved 30 male healthy volunteer participants aged 18–29 years with >5 years of soccer experience. PLR was measured using the NPi-200 pupillometer system before and after performing every 10 headings, up to 30 headings with regular (session 1) and rubber soccer balls (session 2) in separate sessions. The parameters included neurological pupil index (NPi), constriction rate (CH), constriction velocity (CV), and maximum constriction velocity (MCV).

**Results:**

In session 1, CH and MCV significantly decreased compared with the baseline after 30 headings. In session 2, only CH significantly decreased compared with the baseline. CH significantly decreases from the 20th heading onwards in session 1 compared with session 2 (both at 20 and 30 headings; *p* < 0.001). CV significantly decreased after the 30th heading in session 1 compared with session 2 (*p* = 0.038). MCV significantly decreased at the 20th (*p* = 0.037) and 30th (*p* = 0.010) headings in session 1 compared with session 2.

**Conclusion:**

Heading affects PLR, with regular soccer balls causing more significant changes than rubber balls. The use of rubber balls during training may mitigate brain impacts, offering a safer alternative for players.

## 1 Introduction

Soccer is a popular sport, engaging over 265 million people worldwide (1). Soccer heading is associated with repetitive head impacts, which might be linked to adverse effects on cognition, brain microstructure, and brain metabolism (2–5). Indeed, soccer players who have headed the ball frequently can demonstrate image findings similar to those found in traumatic brain injury traumatic brain injury (TBI) (5–7). Furthermore, in the United States, ~30% of concussions in young soccer players have been attributed to heading, highlighting that heading is an important issue for young soccer players (8–11). In response to this issue, the United States Soccer Federation announced a ban on heading for players younger than 10 years in 2016. In 2021, the Japan Football Association similarly introduced guidelines restricting heading for players younger than 15 years, recommending the use of “soft balls (rubber balls)” to mitigate brain impact during practice.

Previously, brain impact associated with heading was assessed using magnetic resonance imaging (MRI) (6, 12) and blood serum measurement (13, 14). Recently, the pupil light reflex (PLR) assessed via pupillometry has been investigated as a potential tool for evaluating TBI (15). Neurological assessment using PLR is becoming more widespread in TBI, post-cardiac arrest, stroke and delirium (16–19). To our knowledge, this is the first study to examine the impact of soccer heading on PLR.

We performed two different experiments to elucidate the influence of heading: (1) measurement of PLR before and after heading and (2) comparison of official Fédération Internationale de Football Association (FIFA) soccer ball with rubber balls.

## 2 Materials and methods

The participants were healthy individuals aged 18–29 years with >5 years of cumulative soccer experience between May 2022 and March 2023. Junior and youth players were not included in this study. Exclusion criteria were a history of neurological diseases (such as concussion, traumatic brain hemorrhage, stroke, brain tumors, and central infections), vestibular dysfunction, non-refractive eye diseases or eye injuries, and psychiatric disorders. We also documented the years of soccer experience, current play category, and primary playing position. Additionally, we also asked if they were soccer players who perceived themselves as proficient in heading. Those who subjectively perceived themselves as confident and experienced in heading were classified as “self-perceived skilled heading.”

### 2.1 PLR measurement

A commercially available pupillometer (NPi-200; Neuroptics Inc., Irvine, CA, USA) was used for PLR analysis. The NPi-200 pupillometer can evaluate eight parameters of the pupil. The neurological pupil index (NPi) values range from 0 to 5, which determined pupillary reactivity, with <3 indicating abnormal reactivity (20). The maximum and minimum size indices were defined as the pupil's largest and smallest diameters (mm), respectively. Constriction rate (CH) was defined as [(maximum pupil diameter (mm) – minimum pupil diameter (mm))/maximum pupil diameter (mm)] × 100 (%). CH ≥15% is considered a normal and brisk response, %CH <15% is a sluggish response, and 0%CH indicates a fixed pupil response (20). Constriction velocity (CV) was defined as the speed at which the pupil constricts following light exposure (mm/s). 1.5 mm/s or higher is considered normal, while <1.0 mm/s is considered pathological (20). Maximum constriction velocity (MCV) and dilation velocity were defined as the maximum speed of pupillary constriction and the speed of pupillary dilation following constriction (mm/s), respectively.

The NPi-200 pupillometer is an FDA-approved Class II medical device and has been validated in previous studies for use in various neurological conditions, including traumatic brain injury, post-cardiac arrest, stroke, and delirium. In particular, Butt et al. demonstrated that NPi values measured with this device contributed to outcome prognostication in TBI patients (15). While the use of NPi in severe neurological disorders is well-established, its role in assessing subtle brain changes, such as those potentially caused by repetitive soccer heading, remains under investigation, and further validation studies are warranted.

### 2.2 Method of heading and measurement of PLR changes

**Figure 1:**
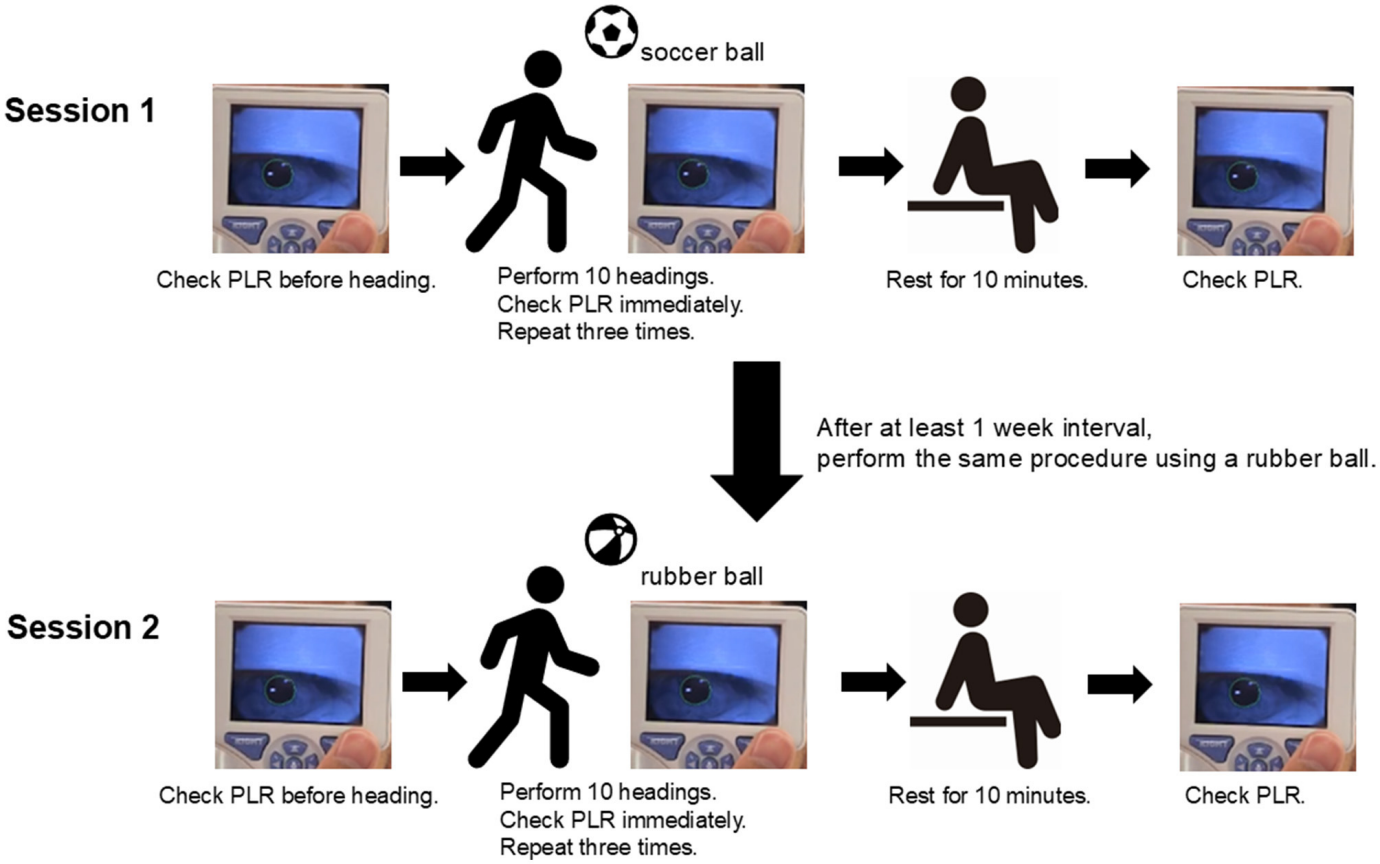
Methods of heading in our study. The participants' pupillary light reflex (PLR) was checked before heading. Immediately after, they performed 10 consecutive standing heading by using a soccer ball, and their PLR was rechecked. This was repeated for three sets. After a 10-min rest, their PLR was rechecked (session 1). After >1 week, the same procedure was repeated using a rubber ball instead of a soccer ball (session 2). PLR, pupillary light reflex.

Before the procedure, baseline PLR measurements were obtained in a controlled environment with consistent lighting conditions. In session 1, we used a regular soccer ball (diameter, 22 cm; 420 g, FIFA official level, 900 hPa) for the heading procedure. The heading method counted one repetition as returning the ball thrown by hand from a distance of 10 m by an assistant by using a standing heading to the chest of the assistant. Subsequently, PLR was measured immediately after completing 10 consecutive headings. Three sets (total, 30 headings) were continuously performed. Subsequently, the participants rested for 10 min before the final PLR measurement. Symptoms associated with heading were checked within 24 h post-test using Post-Concussion Symptom Scale (PCSS), based on the 22 symptoms of somatic, cognitive, and neurobehavioral nature as indicated by the Sports Concussion Assessment Tool 5th edition (SCAT5) (21), to confirm their presence or absence. Session 2 was conducted after at least a 1-week interval. The same participants performed heading but with a rubber ball (diameter, 22 cm; 100 g). PLR measurements before and after heading were performed the same as in session 1. Figure 1 shows the overview of the experiment. Additionally, we also compared PLR changes based on player positions. Defenders (DF) head the ball significantly more frequently (22); hence, we compared PLR changes between DF and attacking players [midfielders (MF) and forwards (FW)]. Goalkeepers (GK) were included in the study. However, they were excluded from position-specific subgroup analyses because they have lower frequency and skills in heading (23).

### 2.3 Statistical analysis

All statistics were reported as mean ± standard error (SE), and statistical analysis was performed using IBM SPSS Statistics 28.0 (IBM, Japan). The Student's *t*-test was used to determine statistically significant differences between the two groups. Repeated measures analysis of variance (ANOVA) (generalized linear model) was used to evaluate repeated changes, and ANOVA was used for comparisons among multiple groups. The Bonferroni method was used for the *post-hoc* test. Pearson's correlation analysis was used to investigate the relationship between years of experience and changes in the PLR. Pearson's correlation coefficient *r* and the associated *p*-value were calculated to assess the strength and significance of the linear relationship. Statistical significance was determined at *p* < 0.05.

The Ethics Committee of the University of Tsukuba Hospital approved this study (R03-290).

## 3 Results

### 3.1 Characteristics of participants

Table 1 shows the characteristics of all participants. Thirty participants were all male. The average age was 23 ± 3 years (range, 18–29 years). The average number of years playing soccer was 15.8 ± 3.2 years. There were 8 DFs (26.7%), 15 MFs (50.0%), 5 FWs (16.7%), and 2 GKs (6.7%). 10 participants (33.3%) were aware of their proficiency in heading.

**Table 1:** Characteristics of participants.

***n***	**30**
Male [number (%)]	30 (100)
Age, year	23 ± 3
Years of soccer experience	15.8 ± 3.2
Players skilled in heading [number (%)]	10 (33.3)
**Position [number (%)]**	
Goal keeper (GK)	2 (6.7)
Defender (DF)	8 (26.7)
Midfielder (MF)	15 (50.0)
Forward (FW)	5 (16.7)
**Playing category [number (%)]**	
College students	24 (80.0)
Amateur player	2 (6.7)
Soccer coach	4 (13.3)

All statistics were reported as mean ± standard error (SE).

### 3.2 Baseline PLR for sessions 1 and 2

The baseline PLR was not significantly different before heading between sessions 1 and 2 [pupil size (3.49 ± 0.09 vs. 3.49 ± 0.08 mm; *p* = 0.973), NPi (4.18 ± 0.06 vs. 4.22 ± 0.06; *p* = 0.621), CH (23.93 ± 1.02% vs. 24.88 ± 1.07%; *p* = 0.542), CV (2.00 ± 0.12 vs. 1.98 ± 0.11 mm/s; *p* = 0.867), and MCV (2.79 ± 0.15 vs. 2.80 ± 0.15 mm/s; *p* = 0.943); Table 2]. All measurement results were within the normal range. Among the 30 participants, 10 were categorized as self-perceived skilled heading. The group included four current or former soccer coaches, 18 university students, and eight working adults.

**Table 2:** Pupillary light reflex of participants before heading.

**Pupillary light reflex before heading**			
**Variables**	**Session 1**	**Session 2**	***p*** **value**
Size(mm)	3.49 ± 0.09	3.49 ± 0.08	0.973
NPi	4.18 ± 0.06	4.22 ± 0.06	0.621
CH (%)	23.93 ± 1.02	24.88 ± 1.07	0.542
CV (mm/sec)	2.00 ± 0.12	1.98 ± 0.11	0.867
MCV (mm/sec)	2.79 ± 0.15	2.80 ± 0.15	0.943

PLR before heading was compared between session 1 and session 2. There were no significant differences between session 1 and session 2 in terms of pupil size. All statistics were reported as mean ± standard error (SE).

PLR, pupillary light reflex; CH, constriction rate; CV, constriction velocity; MCV, maximum constriction velocity.

### 3.3 Changes in PLR after every 10 heading in sessions 1 and 2

In session 1, after 30 headings, CH (−3.10 ± 0.78; *p* = 0.005) and MCV (−0.38 ± 0.11; *p* = 0.033) significantly decreased compared with the baseline. Although NPi (−0.10 ± 0.03; *p* = 0.145) and CV (−0.25 ± 0.09; *p* = 0.168) also decreased compared with the baseline, it did not significantly decrease. All PLRs should be returned to baseline levels after rest.

In session 2, after 30 headings, CH (−2.03 ± 0.57; *p* = 0.013) significantly decreased compared with the baseline. Although NPi (−0.03 ± 0.02; *p* = 1.000), CV (−0.13 ± 0.08; *p* = 1.000), and MCV (−0.13 ± 0.08; *p* = 1.000) also decreased compared with the baseline, it did significantly decrease. NPi did not decline below 3, which is considered critical, in either session.

NPi before and after 30 headings were as follows. In session 1, the mean NPi decreased from 4.18 ± 0.34 to 4.08 ± 0.32 (*p* = 0.145). In session 2, NPi decreased from 4.22 ± 0.29 to 4.19 ± 0.30 (*p* = 1.000).

### 3.4 Comparison of changes between sessions 1 and 2

**Figure 2:**
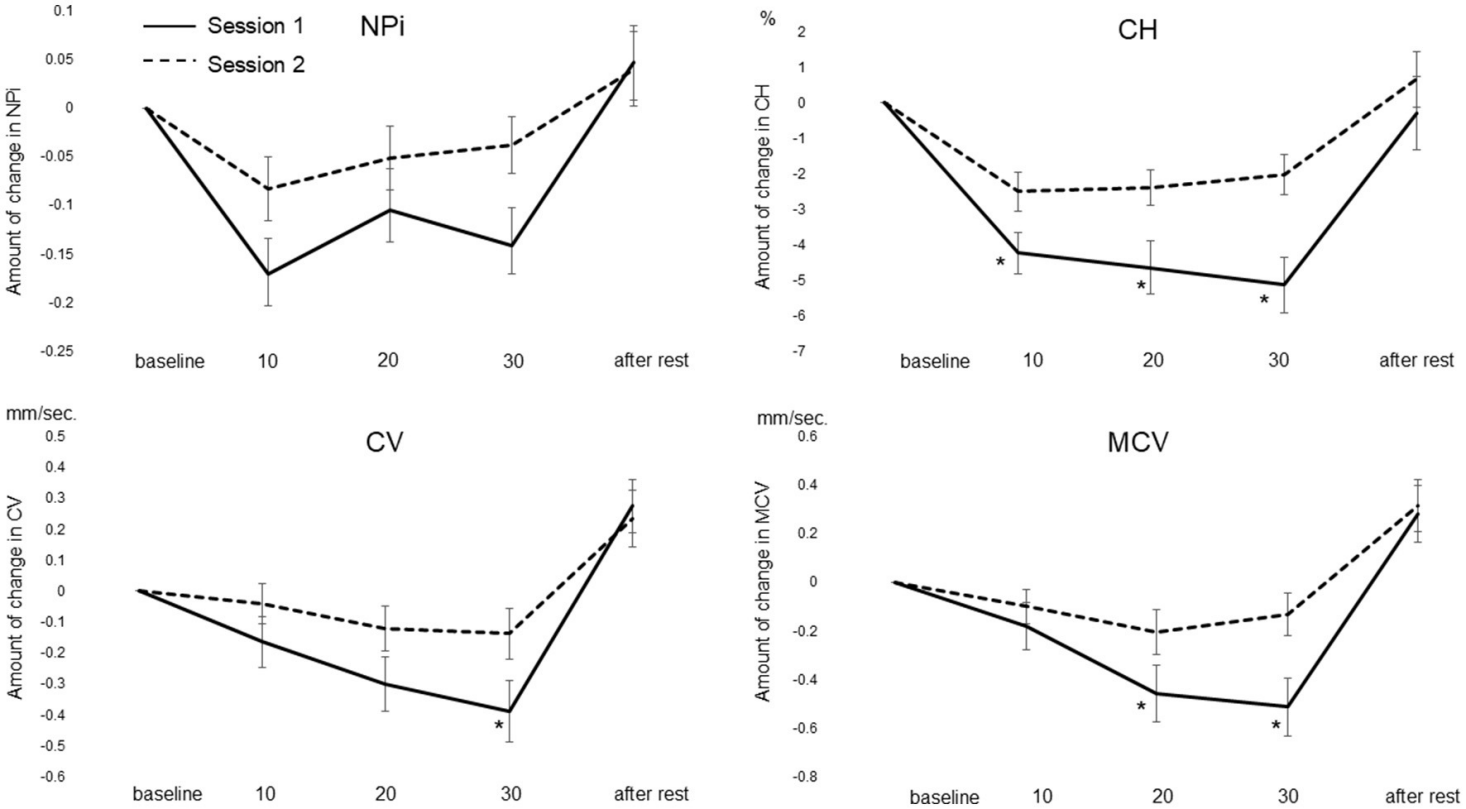
Comparison of the amount of change in pupillary light reflex between sessions 1 and 2. *p < 0.05 (Bonferroni). CH, constriction rate; CV, constriction velocity; MCV, maximum constriction velocity.

Differences between sessions 1 and 2 were also evaluated (Figure 2). After the 30th heading, the NPi in session 1 significantly decreased compared with session 2 (*p* = 0.009). CH significantly decreased from the 20th heading onwards in session 1 compared with session 2 (both at 20 and 30 headers; *p* < 0.001). CV significantly decreased after the 30th heading in session 1 compared with session 2 (*p* = 0.038). MCV significantly decreased at the 20th (*p* = 0.037) and 30th (*p* = 0.010) headings in session 1 compared with session 2. Interval rest between all procedure influenced no difference for all parameters.

To visualize individual variability in change from baseline, paired line plots showing differences in each PLR (CH, CV, MCV, and NPi) were generated for both sessions. These change-based plots are presented as Supplementary Figures S1–S4.

### 3.5 Correlation between years of experience and pupillary changes

**Figure 3:**
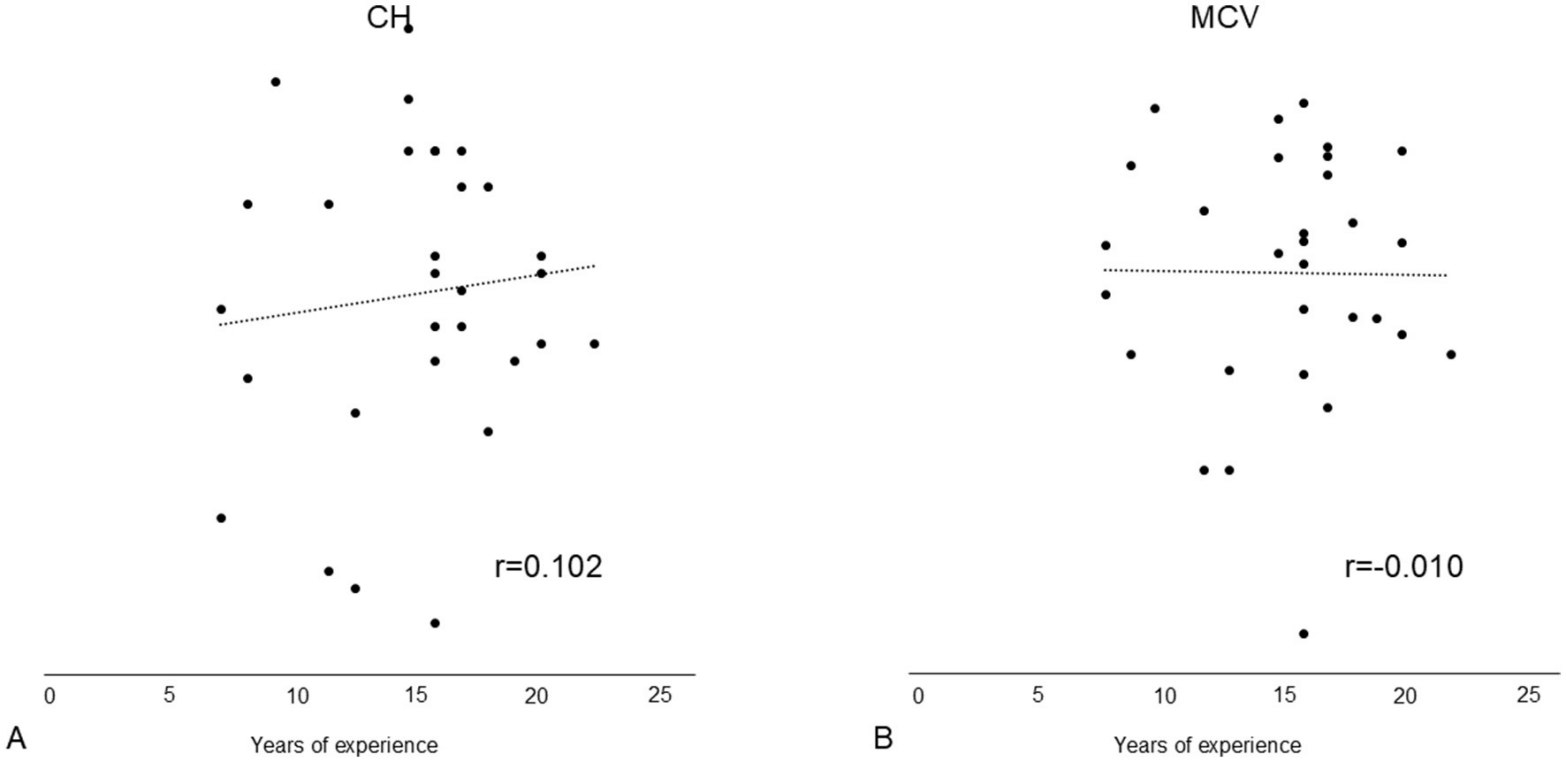
Correlation between years of experience and pupillary light reflex changes in session 1. No significant correlations were found between years of experience and changes in **(A)** CH and **(B)** MCV. CH, constriction rate; MCV, maximum constriction velocity.

We examined the changes in CH and MCV in session 1, which showed larger differences than other indices. No correlations were found between years of experience and changes in CH and MCV (Figure 3).

### 3.6 Difference among soccer positions

We compared the differences in PLR (CH, MCV) among soccer positions. The differences were not significant between the defensive and attacking positions either in CH (−2.31 ± 1.50% vs. −3.39 ± 1.08%, *p* = 0.568) or MCV (−0.21 ± 0.20% vs. −0.47 ± 0.16%, *p* = 0.337; Table 3).

**Table 3:** Pupillary changes by position and players skill.

**(A) Amount of PLR change after heading**			
**Variables**	**DF**	**MF, FW**	***p*** **value**
*n* (%)	8 (26.7)	20 (66.7)	
CH (%)	−2.31 ± 1.50	−3.39 ± 1.08	0.568
MCV (mm/sec)	−0.21 ± 0.20	−0.47 ± 0.16	0.337
**(B) Amount of PLR change after heading**			
**Variables**	**Self-perceived skilled heading**	**Non-self-perceived skilled heading**	***p*** **value**
*n* (%)	10 (33.3)	20 (66.7)	
CH (%)	−3.95 ± 0.99	−2.67 ± 1.00	0.400
MCV (mm/sec)	−0.44 ± 0.13	−0.35 ± 0.15	0.694

A: Changes in CH and MCV by position were compared. Mainly defensive positions (DF) and attacking positions (MF and FW) were compared. No significant differences were observed between the two groups in CH and MCV.

B: Changes in CH and MCV by skill. No significant differences were observed between the two groups in CH and MCV.

All statistics were reported as mean ± standard error (SE).

PLR, pupillary light reflex; DF, defender; MF, midfielder; FW, forward; CH, constriction rate; MCV, maximum constriction velocity.

### 3.7 Pupillary changes based on self-perceived skilled heading

Participants were compared based on their self-perceived skilled heading. No significant differences were observed between the two groups in CH (−3.95 ± 0.99% vs. −2.67 ± 1.00%, *p* = 0.400) or MCV (−0.44 ± 0.13 vs. −0.35 ± 0.15 mm/s, *p* = 0.694; Table 3).

### 3.8 Safety observations

We used the PCSS to assess the symptoms resulting from head injuries after heading. In both Session 1 and Session 2, all participants had a PCSS score of 0, indicating no reported symptoms. Additionally, no adverse events were observed in either session up to 24 h after the procedures.

## 4 Discussion

In severe TBI, changes in PLR can precede increases in intracranial pressure, and changes in NPi and CH may serve as biomarkers of increasing intracranial pressure (24–26). Some studies showed decreased CH and MCV in asymptomatic cases of TBI with rotational acceleration (27). Our study was the first to evaluate PLR changes caused by soccer heading. We also investigated whether different ball types in soccer heading could have different impacts on the brain.

We found significant decreases in CH and MCV after 30 times of headings with a regular soccer ball compared with heading using a rubber ball. Additionally, changes in PLR due to heading were not dependent on the player's position, years of experience, or self-reported proficiency in heading.

One study has reported methods for evaluating brain damage associated with heading in soccer, which identified changes in white matter structure in MRI-based assessments after long-term exposure to heading (4). However, these studies had small sample sizes. Moreover, long-term cumulative exposure to heading was associated with neurodegenerative change, similar to TBI (6, 28). In professional soccer players, serum S-100B, a biomarker of brain tissue damage, increased after heading, and this increase may be correlated with the number of headings (13, 29–31). Therefore, if we could monitor brain impact from the time of training, safer training methods of heading can be implemented. However, imaging and biomarker evaluation methods require specialized equipment and can be invasive, making them unsuitable as universal evaluation methods.

Depending on the situation, heading can involve significant rotational acceleration, which may contribute to brain impact. CH and MCV decrease in players with sustained concussion or even those not diagnosed as concussion due to rotational acceleration in American football (27), recent soccer-specific studies using instrumented mouthguards have also demonstrated significant rotational head acceleration and brain strain during heading (32). High cumulative rotational acceleration has been associated with microstructural changes in the brain (33), and even mild cases involving rotational head movements can result in changes in CH and MCV.

In addition to the other PLR parameters, we evaluated changes in NPi before and after heading. The absolute changes were small and all values remained within the normal range (>3). This suggests that heading may induce minor physiological alterations in pupillary response, but without evidence of clinically relevant impairment in global pupillary reactivity.

It should be noted that NPi is a composite metric generated by a proprietary algorithm that integrates multiple pupillary parameters. While it has been widely used in neurocritical care and shown to have prognostic value in conditions such as traumatic brain injury, cardiac arrest, and stroke, the exact physiological interpretation of small NPi fluctuations remains unclear. Thus, although the observed NPi changes were not clinically alarming in this study, future research is needed to further elucidate its sensitivity and specificity in sports-related brain impact.

Conversely, MCV did not change in headings with a rubber ball (session 2) in this study. Heading with a lighter ball can reduce the rotational acceleration of the head, and heading with a rubber ball might have led to no changes in MCV. A negative correlation was found between the mass of the head and neck and the acceleration experienced during heading, the larger the mass, the smaller the acceleration (34). We excluded junior or youth players, known to have a higher head mass ratio relative to their body size, which might mean insufficient neck muscle strength to support the head during heading, potentially leading to greater acceleration (35). Thus, heading training with lighter balls, such as rubber balls might reduce the brain impact.

## 5 Limitations

This study has several limitations. The implications of PLR changes in mild TBI remain unclear. Further investigations should clarify the mechanisms by which head impact affects PLR.

Additionally, all participants were male, thus, we lacked female data. The number of female soccer players has been increasing, with ~13.3 million female players worldwide in 2019 (36). FIFA has set a goal to further increase this number. Therefore, information on heading in female players is significant for the future development of soccer (36). Because female players head the ball less frequently than male players but may experience greater impact (37, 38), additional research is necessary before these results can be applied to female players.

We were unable to include a control group due to practical constraints. The absence of a comparator condition limits the ability to distinguish the specific effects of heading from those of general physical exertion or other potential confounding factors. We observed that soccer heading was associated with changes in PLR, suggesting a potential impact on the brain. While the clinical significance of these changes remains to be fully understood, our findings indicate that using a rubber ball may help mitigate this effect.

Furthermore, we did not measure the velocity of the ball during the heading sessions. Although we standardized the heading drills by using the same throwing method and distance for all participants, the absence of quantitative ball speed data limits our ability to precisely compare the impact intensity with that encountered during actual competitive play. This restricts the generalizability of our findings to real-game conditions, where heading velocity may be higher and more variable.

Future research should aim to incorporate study designs that better replicate real-world football conditions and include appropriate control groups to account for potential confounding variables. Additionally, methodological guidelines should be considered to enhance both the ecological and external validity of future studies (39, 40).

## 6 Conclusion

Heading affects PLR (CH and MCV), with regular soccer balls causing more significant changes than rubber balls. The use of rubber balls during training may mitigate brain impacts, offering a safer alternative for players.

## Data Availability

The raw data supporting the conclusions of this article will be made available by the authors, without undue reservation.

## Supplementary material

Supplementary Figure S1 Individual changes in CH (change from baseline) before and after 30th headings in session 1 and session 2. Each line represents the change from baseline (before heading = 0) to after 30th headings for an individual participant. This figure illustrates individual-level differences in the change in constriction percentage (CH) following heading. CH, constriction percentage.

Supplementary Figure S2 Individual changes in CV (change from baseline) before and after 30th headings in session 1 and session 2. Each line represents the change from baseline (before heading = 0) to after 30th headings for an individual participant. This figure illustrates individual-level differences in the change in constriction velocity (CV) following heading. CV, constriction velocity.

Supplementary Figure S3 Individual changes in MCV (change from baseline) before and after 30th headings in session 1 and session 2. Each line represents the change from baseline (before heading = 0) to after 30th headings for an individual participant. This figure illustrates individual-level differences in the change in maximum constriction velocity (MCV) following heading. MCV, maximum constriction velocity.

Supplementary Figure S4 Individual changes in NPi (change from baseline) before and after 30th headings in session 1 and session 2. Each line represents the change from baseline (before heading = 0) to after 30th headings for an individual participant. This figure illustrates individual-level differences in the change in neurological pupil index (NPi) following heading. NPi, neurological pupil index.
