# Supplementary material for: Assessment of the brain impact of soccer heading using pupillary light reflex
Source: Front Neurol. 2025 May 27;16:1603033. doi: 10.3389/fneur.2025.1603033 (PMC12148879; doi:10.3389/fneur.2025.1603033)
Supplement: Supplementary file 1 [file Table_1.docx]

Supplementary Table S1

Pupillary light reflex parameters before and after heading in sessions

| Pupillary Light Reflex before and after heading | | | | |
| --- | --- | --- | --- | --- |
| variables | Session 1 before heading | Session 1 after heading | Session 2 before heading | Session 2 after heading |
| Size(mm) | 3.49±0.09 | 3.36 ± 0.08 | 3.49±0.07 | 3.37 ± 0.08 |
| NPi | 4.18±0.06 | 4.06 ± 0.06 | 4.22±0.05 | 4.19 ± 0.06 |
| CH(%) | 23.93±1.02 | 20.83 ± 1.07 | 24.88±1.16 | 22.85 ± 1.28 |
| CV(mm/sec.) | 2.00±0.12 | 1.77 ± 0.11 | 1.98±0.08 | 1.85 ± 0.11 |
| MCV(mm/sec.) | 2.79±0.15 | 2.42 ± 0.15 | 2.80±0.12 | 2.68 ± 0.15 |

Pupillary light reflex (PLR) parameters before and after heading in Session 1 (regular ball) and Session 2 (rubber ball). All statistics were reported as mean ± standard error (SE).

PLR: Pupillary light reflex, CH: constriction rate, CV: constriction velocity, MCV: maximum constriction velocity
