# Supplementary figures and images for: Assessment of the brain impact of soccer heading using pupillary light reflex
Source: Front Neurol. 2025 May 27;16:1603033. doi: 10.3389/fneur.2025.1603033 (PMC12148879; doi:10.3389/fneur.2025.1603033)

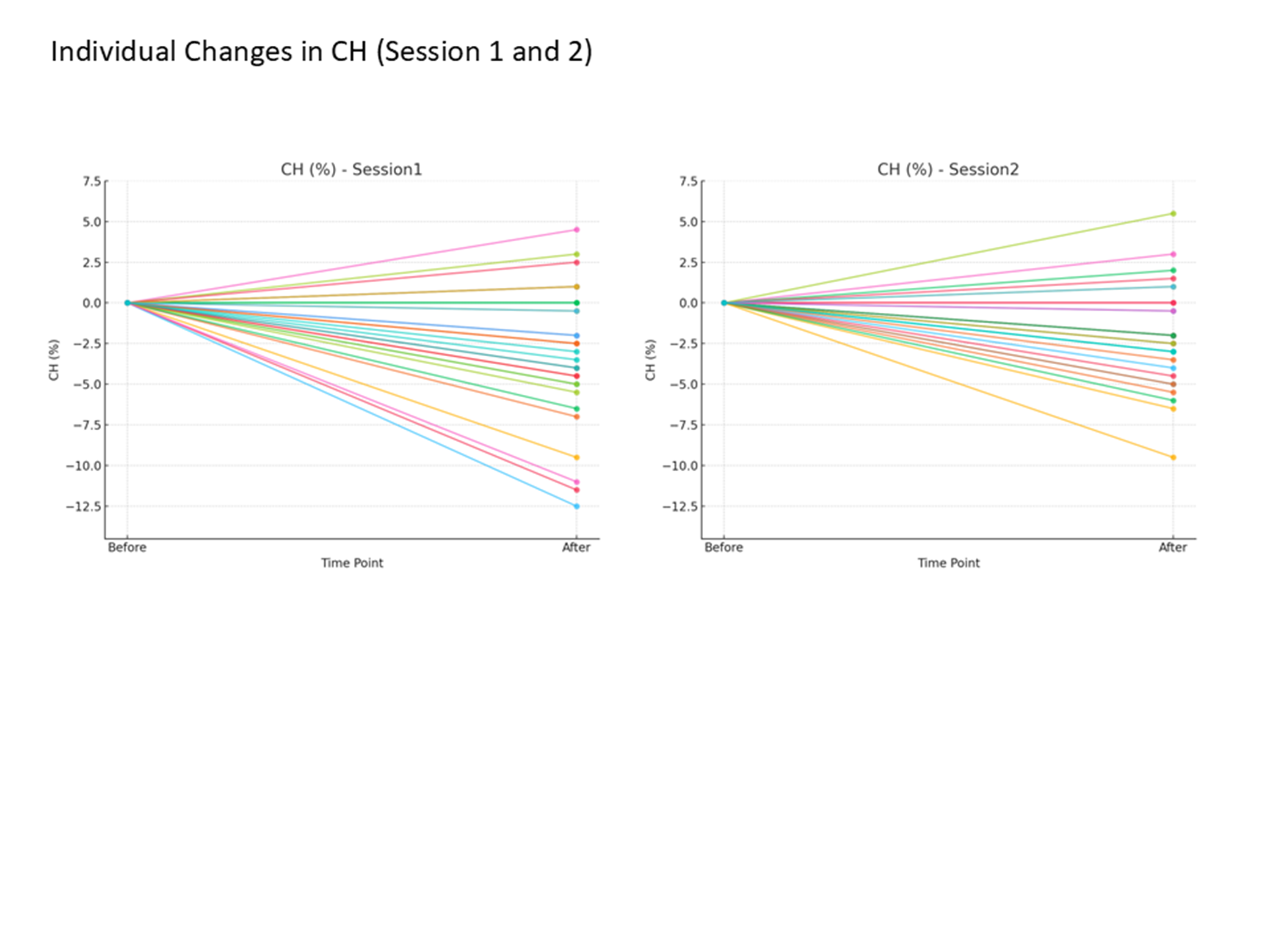

Supplement: Supplementary Figure S1 — Individual changes in CH (change from baseline) before and after 30th headings in session 1 and session 2. Each line represents the change from baseline (before heading = 0) to after 30th headings for an individual participant. This figure illustrates individual-level differences in the change in constriction percentage (CH) following heading. CH, constriction percentage. [file Image_1.tif]

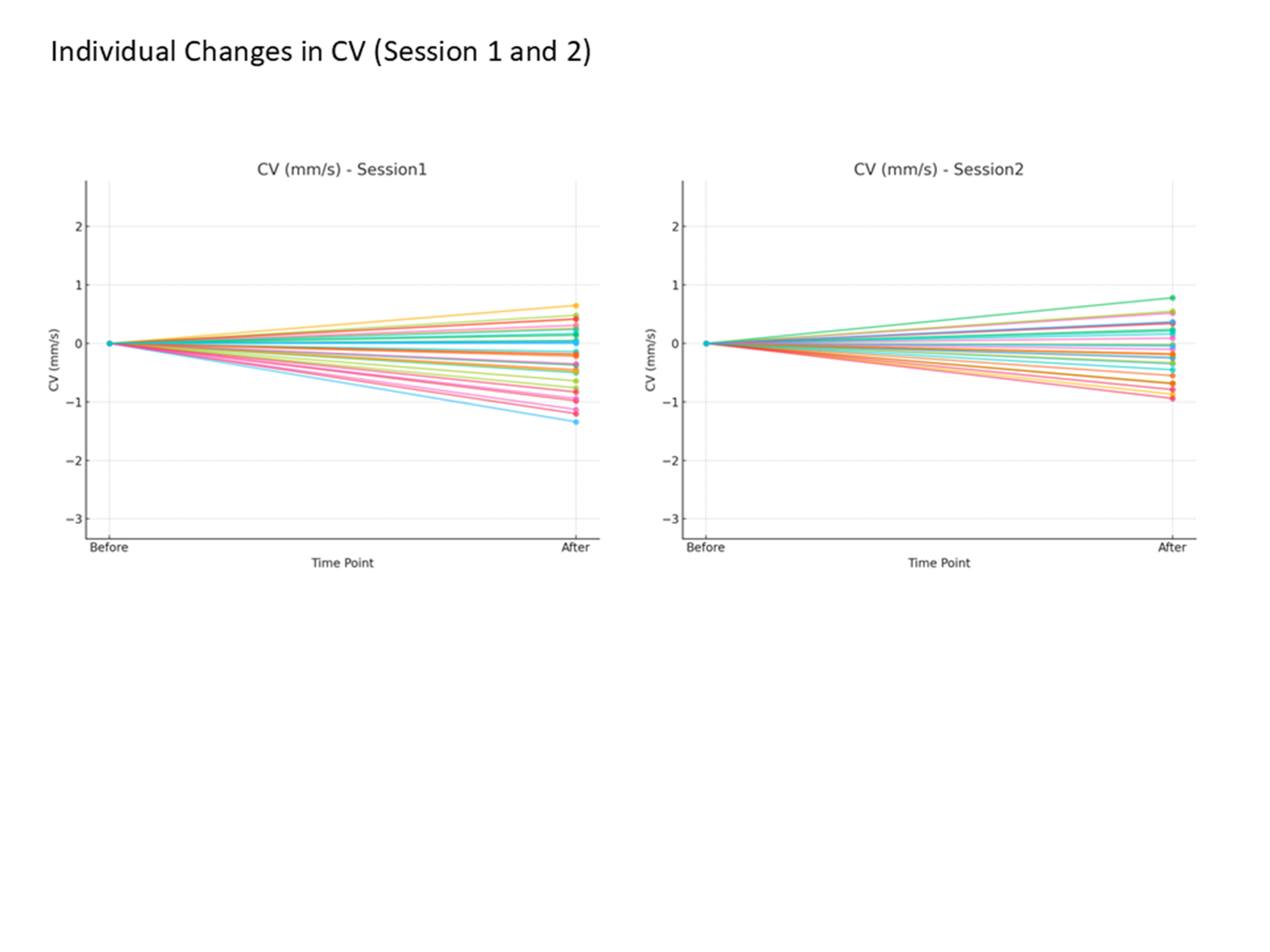

Supplement: Supplementary Figure S2 — Individual changes in CV (change from baseline) before and after 30th headings in session 1 and session 2. Each line represents the change from baseline (before heading = 0) to after 30th headings for an individual participant. This figure illustrates individual-level differences in the change in constriction velocity (CV) following heading. CV, constriction velocity. [file Image_2.tif]

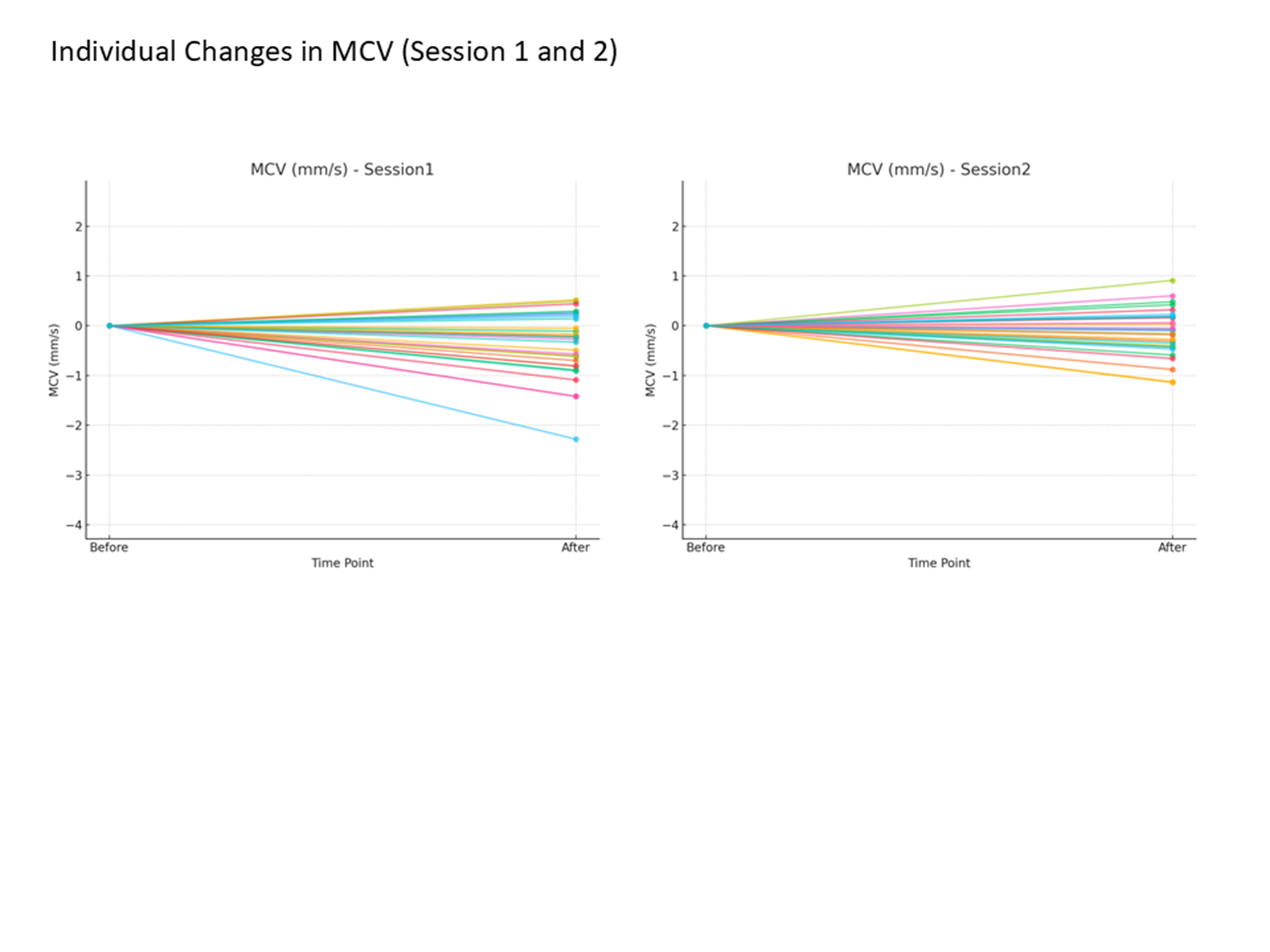

Supplement: Supplementary Figure S3 — Individual changes in MCV (change from baseline) before and after 30th headings in session 1 and session 2. Each line represents the change from baseline (before heading = 0) to after 30th headings for an individual participant. This figure illustrates individual-level differences in the change in maximum constriction velocity (MCV) following heading. MCV, maximum constriction velocity. [file Image_3.tif]

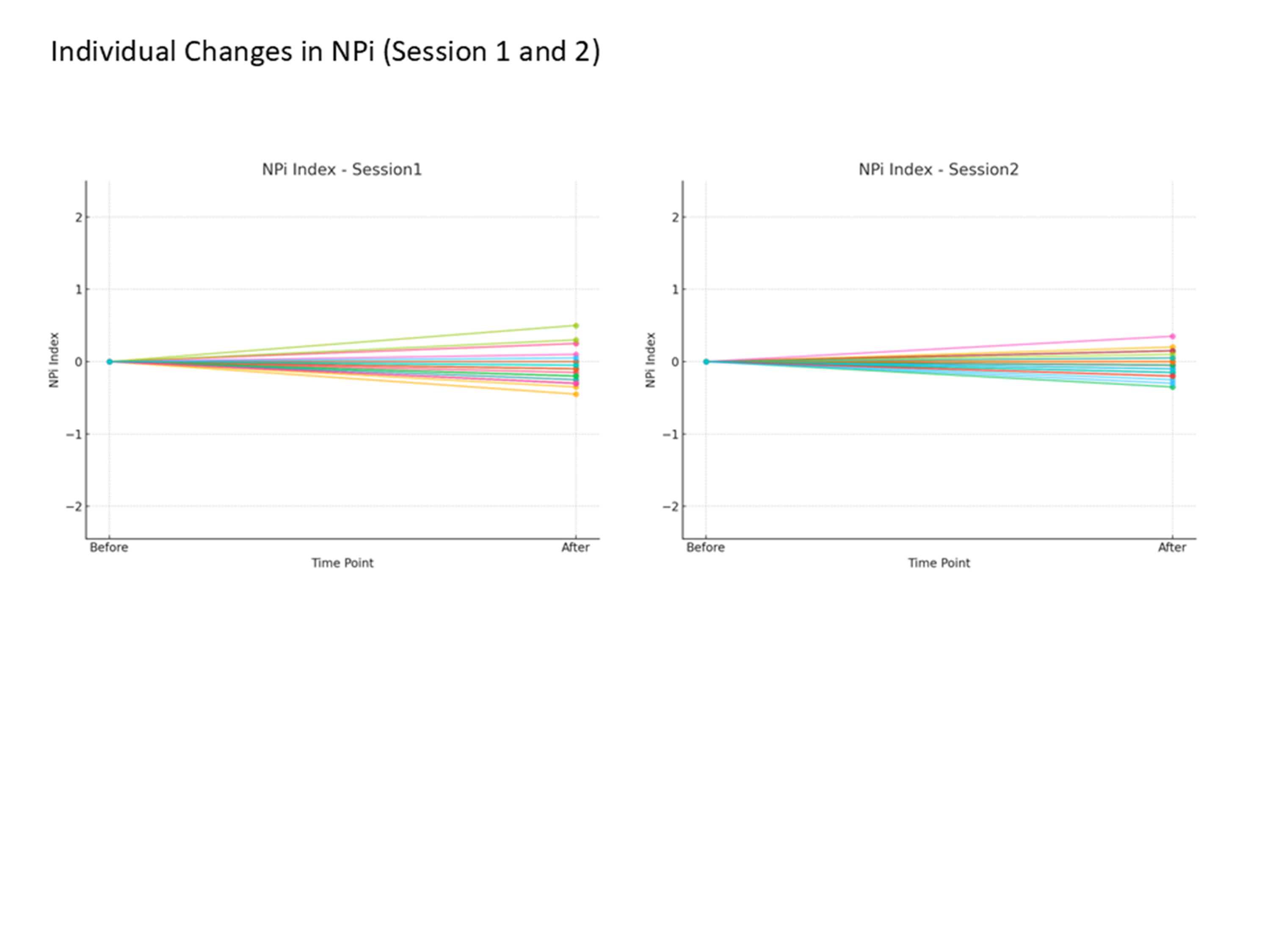

Supplement: Supplementary Figure S4 — Individual changes in NPi (change from baseline) before and after 30th headings in session 1 and session 2. Each line represents the change from baseline (before heading = 0) to after 30th headings for an individual participant. This figure illustrates individual-level differences in the change in neurological pupil index (NPi) following heading. NPi, neurological pupil index. [file Image_4.tif]
